# Validation of metagenomic next-generation sequencing of bronchoalveolar lavage fluid for diagnosis of suspected pulmonary infections in patients with systemic autoimmune rheumatic diseases receiving immunosuppressant therapy

**DOI:** 10.3389/fmed.2023.1161661

**Published:** 2023-07-06

**Authors:** Sichun Wen, Siqi Peng, Xuejiao Hu, Nan Jiang, Bohou Li, Boxi Chen, Shuting Deng, Ye Yuan, Qiong Wu, Yiming Tao, Jianchao Ma, Sijia Li, Ting Lin, Feng Wen, Zhuo Li, Renwei Huang, Zhonglin Feng, Chaosheng He, Wenjian Wang, Xinling Liang, Wei Shi, Lixia Xu, Shuangxin Liu

**Affiliations:** ^1^School of Medicine, South China University of Technology, Guangzhou, Guangdong, China; ^2^Department of Nephrology, Guangdong Provincial People's Hospital (Guangdong Academy of Medical Sciences), Southern Medical University, Guangzhou, China; ^3^The Second School of Clinical Medicine, Southern Medical University, Guangzhou, China; ^4^Laboratory Medicine, Guangdong Provincial People's Hospital (Guangdong Academy of Medical Sciences), Southern Medical University, Guangzhou, China

**Keywords:** metagenomic next-generation sequencing, systemic autoimmune rheumatic diseases, immunosuppressants, bronchoalveolar lavage fluid, pulmonary infection, antibiotics

## Abstract

**Background:**

The accuracy and sensitivity of conventional microbiological tests (CMTs) are insufficient to identify opportunistic pathogens in patients with systemic autoimmune rheumatic diseases (SARDs). The study aimed to assess the usefulness of metagenomic next-generation sequencing (mNGS) vs. CMTs for the diagnosis of pulmonary infections in patients with SARDs receiving immunosuppressant therapy.

**Methods:**

The medical records of 40 patients with pulmonary infections and SARDs treated with immunosuppressants or corticosteroids were reviewed retrospectively. Bronchoalveolar lavage fluid (BALF) samples were collected from all patients and examined by mNGS and CMTs. Diagnostic values of the CMTs and mNGS were compared with the clinical composite diagnosis as the reference standard.

**Results:**

Of the 40 patients included for analysis, 37 (92.5%) were diagnosed with pulmonary infections and 3 (7.5%) with non-infectious diseases, of which two were considered primary diseases and one an asthma attack. In total, 15 pathogens (7 bacteria, 5 fungi, and 3 viruses) were detected by CMTs as compared to 58 (36 bacteria, 12 fungi, and 10 viruses) by mNGS. Diagnostic accuracy of mNGS was superior to that of the CMTs for the detection of co-infections with bacteria and fungi (95 vs. 53%, respectively, *p* < 0.01), and for the detection of single infections with fungi (97.5 vs. 55%, respectively, *p* < 0.01). Of the 31 patients diagnosed with co-infections, 4 (12.9%) were positive for two pathogens and 27 (87.1%) for three or more. The detection rate of co-infection was significantly higher for mNGS than CMTs (95 vs. 16%, respectively, *p* < 0.01).

**Conclusion:**

The accuracy of mNGS was superior to that of the CMTs for the diagnosis of pulmonary infections in patients with SARDs treated with immunosuppressants. The rapid diagnosis by mNGS can ensure timely adjustment of treatment regimens to improve diagnosis and outcomes.

## Introduction

Systemic autoimmune rheumatic diseases (SARDs) are a group of autoimmune-mediated diseases characterized by the overproduction of autoantibodies. The incidence of SARDs has continued to increase in recent years (1). The propensity for pulmonary infection of patients with SARDs is both an inherent form of disease-related immune dysregulation and acquired by use of immunosuppressants (2). Treatment of SARDs involves using immunosuppressants or immunomodulatory drugs, such as mycophenolate mofetil, azathioprine, and tacrolimus (3). However, immunosuppression and severe activity of the underlying disease often lead to infection by various opportunistic pathogens (4, 5), such as Cryptococcus novelis, which can cause fatal meningoencephalitis in immunocompromised individuals, and Pneumocystis jirovecii, which can cause pneumonia after hematopoietic stem cell and solid organ transplantation and is especially problematic in patients receiving immunosuppressants. Due to the abundance of potential pathogens and the possibility that the symptoms of the primary disease or the treatment regimen may hide an actual pulmonary infection, diagnosis of this population is often challenging (6).

Accurate diagnosis and effective treatment of infections often require testing of pathogens for drug sensitivity. Conventional microbiological tests (CMTs), such as culture-based detection assays, immunological analysis, and polymerase chain reaction (PCR), are commonly used for clinical diagnosis of Pneumocystis jirovecii pneumonia (PJP) (7). However, CMTs are limited by insufficient sensitivity and speed for accurate and rapid identification of pathogens in samples from immunocompromised patients (8). Thus, alternative methods, such as multiplex real-time quantitative fluorescent PCR, biosensors, and metagenomic next-generation sequencing (mNGS), are potentially superior diagnostic options for this patient population. mNGS is a high-throughput nucleic acid sequencing technology that has been widely applied to detect various pathogens (9–13). The advantages of mNGS include shorter detection times and accurate detection of multiple pathogens simultaneously by DNA or mRNA sequencing of clinical samples (14). While mNGS has been used to diagnose pneumonia in immunocompromised patients, with advantages in pathogen detection, particularly in fungal and co-infections, studies exploring its potential application in patients with SARDs remain limited (15, 16). The present study aimed to compare the diagnostic accuracy of mNGS vs. CMTs for the detection of pulmonary infections in immunocompromised patients.

## Materials and methods

The cohort of this retrospective study included 40 patients with SARDs and suspected pulmonary infections who were admitted to Guangdong Provincial People’s Hospital (Guangzhou, China) from April 2021 to July 2022 and met the following inclusion criteria: (i) confirmed diagnosis of SARDs, included systemic lupus erythematosus (SLE), systemic vasculitis, rheumatoid arthritis(RA), dermatomyositis(DM), primary Sjögren’s syndrome, immunoglobulin G4-related disease, Adult-onset Still’s disease (AOSD), and mixed connective tissue disease (MCTD). (ii) long-term use of immunosuppressants or corticosteroids, >0.5 mg/kg/day, >1 month; (iii) prior bronchoscopy and collection of bronchoalveolar lavage fluid (BALF); (iv) pathogen detection by mNGS and CMTs with bacterial and fungal smears and cultures; and (v) suspected pulmonary infection confirmed by radiographic images, CT signs included ground glass opacity (GGO), nodules, inflatable sign, parenchymal opacification, reticular or linear shadow, interstitial pneumonia (17).

### Data collection

Demographic and clinical data, including age, sex, type of rheumatic disease, use of steroids and immunosuppressants, underlying illness, chest images, results of CMTs and mNGS, changes to antibiotic therapy, and disease regression data, were obtained from electronic medical records.

### CMTs

Blood and BALF samples were obtained from all patients. All bronchoalveolar lavage procedures were performed following standard safety protocols. Cultures and smears of the BALF and sputum samples were immediately prepared. All blood samples were immediately assayed by PCR for detection of human immunodeficiency virus (HIV), Epstein–Barr virus (EBV), cytomegalovirus (CMV), and herpes simplex virus in addition to serological analysis of immunoglobulin G, galactomannan, and *Mycobacterium tuberculosis* infection.

### mNGS detection using BALF samples

The mNGS procedure for BALF samples included nucleic acid extraction, library construction, sequencing, and bioinformatics analysis. When analyzing and extracting respiratory DNA samples, the Jinshi MicroDNA Kit is mainly used for extraction, followed by DNA fragmentation, splicing, biomolecular labeling (barcode) amplification, and onboard sequencing. The quality of the library is achieved through Qubit® one × dsDNA HS assay was used for evaluation, and then evaluated using Qubit4.0 fluorometer. After the library was quantified by real-time PCR, the MGISEQ-200RS high-throughput sequencing platform was used to sequence the library by the shotgun method. It usually takes 3 days to complete the mNGS analysis and publish the report. The mNGS results were interpreted in reference to the criteria used in previous reports of mNGS for the identification of clinically relevant microorganisms (CRMs) with the Beijing Genomics Institute NGS (BGISEQ-500) platform (15, 18). Bacteria (mycobacteria excluded), fungi (molds excluded), viruses, and parasites with relative abundances at the species level of >30% were considered CRMs. Mycobacteria with a strict mapping read number (SMRN) at the species level of >3 and those with evidence of pulmonary pathogenicity and a SMRN at the species level of >10 were also considered CRMs.

### Clinical composite diagnosis as the reference standard

Clinical composite diagnostic criteria were used to identify the pathogens, as determined by two experienced clinicians based on epidemiology, clinical presentation, treatment outcome, laboratory findings, and chest radiology. A true positive was defined as consistency between the results of mNGS and CMTs, while a false positive was defined as detection by mNGS, but not considered pathogenic by the gold standard. If two experts could not reach an agreement, in-depth discussions were held with a third expert.

### Statistical analysis

The accuracy, sensitivity, specificity, positive predictive value, and negative predictive value (NPV) of the data were calculated and compared. The chi-square test and Fisher exact test were conducted to identify differences between the results of mNGS and CMTs. All statistical analyses were performed with IBM SPSS Statistics for Windows, version 26.0 (IBM Corporation, Armonk, NY, USA). A probability (*p*) value <0.05 was considered statistically significant.

## Results

### General characteristic

The study cohort comprised 40 patients (15 males and 25 females; mean age, 50.82 years) with a confirmed pulmonary infection. The clinical characteristics of the patients are shown in Table 1. All 40 patients had SARDs, which included 22 (55%) with either systemic lupus erythematosus or systemic vasculitis, and were treated with immunosuppressants or hormone therapy. The most commonly used immunosuppressants were cyclophosphamide (14/40, 35%), mycophenolate mofetil (10/40, 25%), and methotrexate (9/40, 22.5%).

**Table 1:** The baseline of patients with pneumonia infection.

Characteristic	Clinical value
Age(avg, y)	50.82 ± 16.06
Sex	
Male	37.5% (15/40)
Female	62.5% (25/40)
Primary disease	
SLE	30.0% (12/40)
Systemic vasculitis	25.0% (10/40)
Sjogren’s syndrome	12.5% (5/40)
Dermatomyositis	15.0% (6/40)
Other disease	17.5% (7/40)
Immunosuppressant	
Azathioprine	12.5% (5/40)
Cyclophosphamide	35% (14/40)
Mycophenolatemofetil	25% (10/40)
Methotrexate	22.5% (9/40)
Biological agents	10% (4/40)
Leflunomide	2.5% (1/40)
Cyclosporine	15% (6/40)
Corticosteroid	95% (38/40)
Diabetes	
Yes	7.5% (3/40)
No	92.5% (37/40)
Symtom	
Fever	37.5% (15/40)
Cough	25% (10/40)
Anhelation	22.5% (9/40)
Diarrhea	7.5% (3/40)
Asymptomatic	7.5% (3/40)
Lab tests	
WBC(×109/L)	8.63 ± 0.62
Neutrophils%	82.25 (69.58, 89.20)
Lymphocyte(×109/L)	0.83 (0.52,1.18)
Lymphocyte%	0.09 (0.06, 0.18)
Hb(g/L)	101.60 ± 4.08
Erythrocyte sedimentation rate(mm/h)	35.50 (7.25, 61.25)
CRP(mg/L)	22.20 (4.53, 46.53)
PCT(ng/L)	0.09 (0.05, 0.47)
CREA(μmol/L)	77.15 (47.03, 117.25)
ALT(U/L)	16.00 (10.25, 34.50)
Outcomes	
Improved	62.5% (25/40)
Deteriorate	12.5% (5/40)
Loss to follow-up	22.5% (9/40)
Died	2.5% (1/40)

SLE, systemic lupus erythematosus; WBC, white blood cell; Hb, hemoglobin; CRP, C-reaction protein; PCT, procalcitonin; CREA, creatinine; ALT, alanine aminotransferase.

### Diagnostic performance of mNGS vs. CMTs

**Figure 1:**
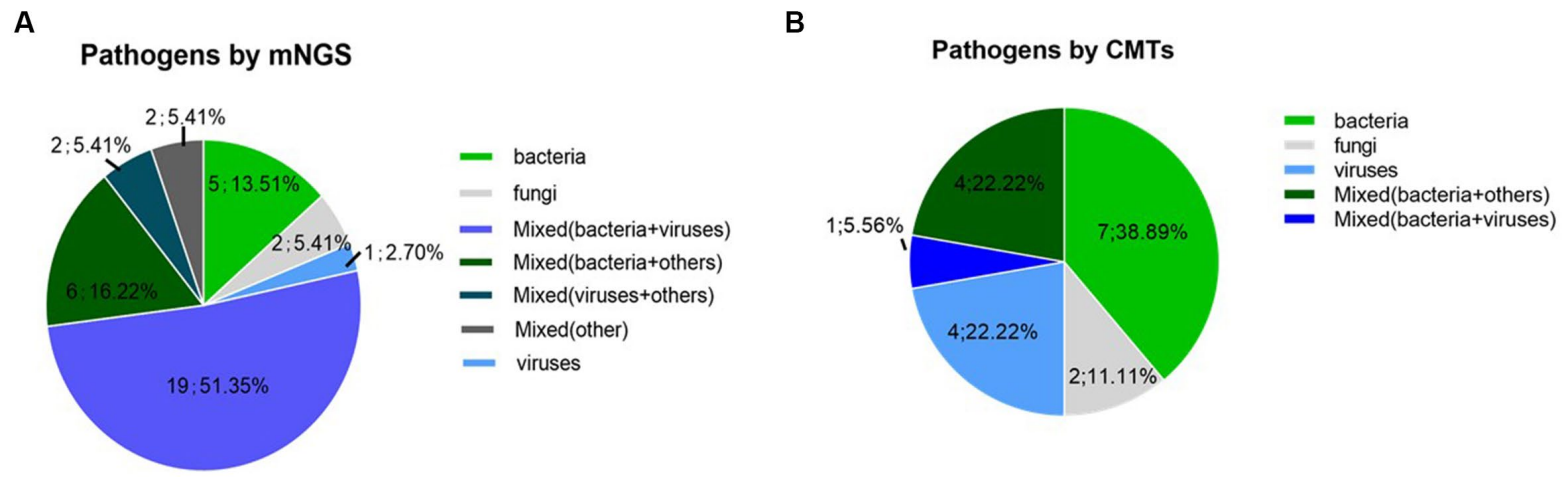
Pathogen detection using mNGS and conventional methods **(A)** Detection of mNGS, **(B)** Detection of conventional methods.

**Figure 2:**
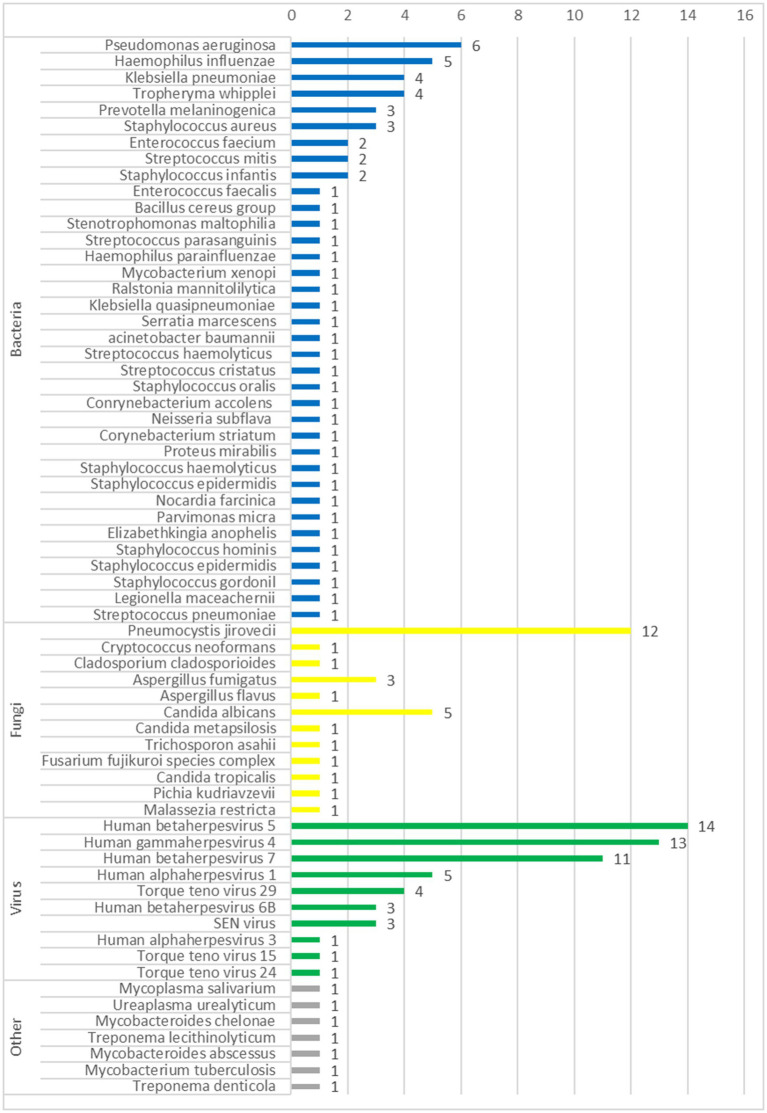
The number of pathogens of all cases detected by mNGS.

The pathogens identified by CMTs and mNGS among the 40 patients are shown in Figure 1. Of the 40 patients, 37 (92.5%) had confirmed pulmonary infections by NGS. The isolated pathogens included 36 bacterial, 12 fungal, and 10 viral species. The most common bacterial, fungal, and viral pathogens were *Pseudomonas aeruginosa*, P. jirovecii, and CMV, respectively (Figure 2). The positivity rate of CMTs was significantly lower than that of mNGS (42.5 vs. 92.5%, respectively, *p* < 0.01). The CMTs detected seven bacteria, five fungi (two Cryptococcus), and three viral species.

### Consistency of mNGS with CMTs

**Figure 3:**
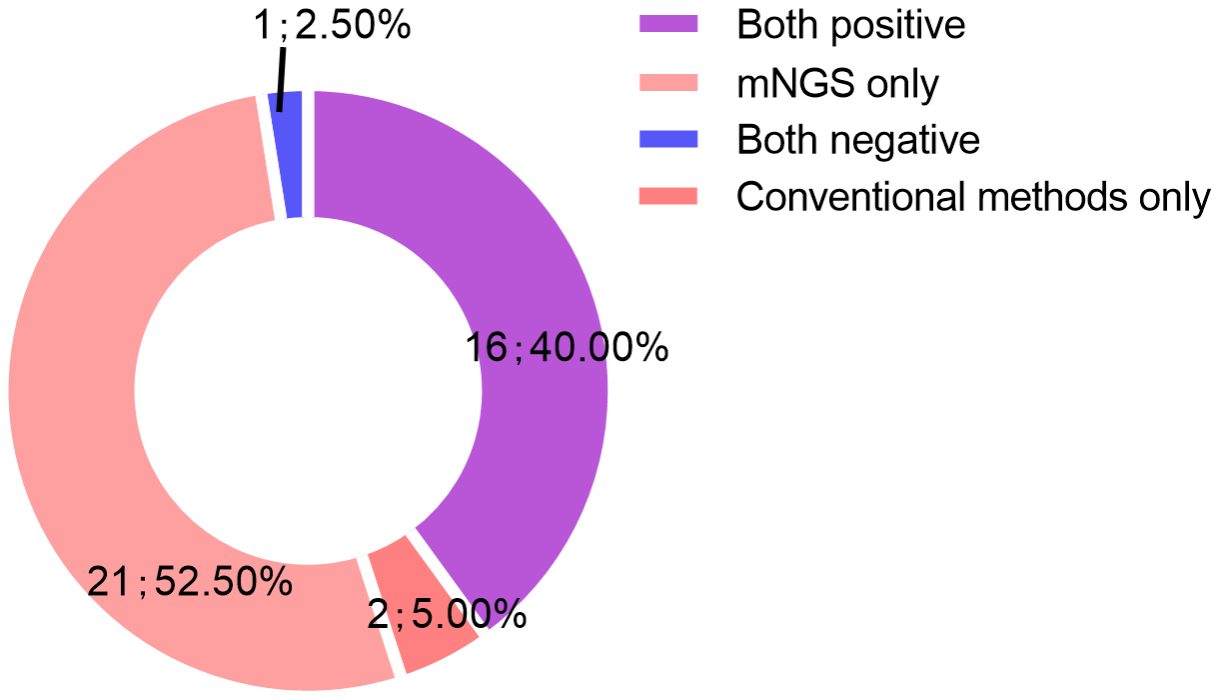
Consistency between mNGS and CMTs.

Of the 40 cases, 16 (40%) were positive for pathogens by both mNGS and CMTs, 21 (52.5%) by only mNGS, and 2 (5%) by only CMTs, while 1 (2.5%) was negative by both mNGS and CMTs (Figure 3). Of 15 double-positive cases, 1 (6.7%) had an exact match between mNGS and CMTs, 2 (13.3%) had a clear mismatch, and 12 (80%) were partial matches, meaning that at least one pathogen was detected by both the CMTs and mNGS.

### Diagnostic sensitivity and specificity of mNGS vs. CMTs

For the identification of bacteria, the diagnostic sensitivity and specificity of the CMTs and mNGS were 35%(11/31)/97%(30/31) and 89%(8/9)/89%(8/9), respectively. Notably, the diagnostic accuracy of mNGS for the identification of bacteria was significantly greater than that of the CMTs (95% vs. 53%, respectively, *p* < 0.01). For the detection of fungi, the diagnostic sensitivity and specificity of the CMTs and mNGS were 23%/95% and 94%/100%, respectively. Similarly, the diagnostic accuracy of mNGS for the identification of fungi was significantly greater than that of the CMTs (97.5 vs. 55%, respectively, *p* < 0.01).

### Performance of mNGS vs. CMTs for detection of co-infections

Of the 40 patients, 4 (10%) were infected with two pathogens and 27 (67.5%) with three or more. The co-infection detection rate was significantly higher with mNGS than with the CMTs (95 vs. 16%, respectively, *p* < 0.01), and mNGS detected a greater variety of pathogens. Coinfections with fungal-viral-bacterial and bacterial-viral species were the most common. The most common co-infection pathogens involved were P. jirovecii (12/40, 30%) and CMV (13/40, 32.5%).

### Diagnostic accuracy of mNGS vs. CMTs

Of the 40 patients, 37 (92.5%) were diagnosed with pneumonia and 3 (7.5%) with non-infectious diseases, of which two were considered primary diseases, and one was an asthma attack. Of the 37 patients diagnosed with pneumonia, 2 (5.4%) had negative mNGS results. All 3 (100%) patients diagnosed with non-infectious diseases had false positive mNGS results, demonstrating sensitivity of 95% (95% confidence interval [CI] = 80–99%), specificity of 33% (95% CI = 1.8–87%), NPV of 33%, and accuracy of 90% (95% CI = 81–99%). In contrast, CMTs misdiagnosed infection in 19 (51.4%) of 37 pneumonia patients, which included one false positive for *P. aeruginosa* infection, demonstrating sensitivity of 47% (95% CI = 30–64%), specificity of 75% (95% CI = 22–99%), NPV of 14%, and accuracy of 50% (95% CI = 35–65%). The NPV and diagnostic accuracy of mNGS were superior to those of the CMTs (Table 2).

**Table 2:** The sensitivity and specificity of mNGS and CMTs.

Assay	Sensitivity, % (95%Cl)	Specificity, % (95%Cl)	PPV (%)	NPV (%)	PLR	NLR
mNGS	94.59 (0.80 ~ 0.99)	33.33 (0.02 ~ 0.87)	94.59	33.33	1.42	0.16
CMTs	33.33 (0.19 ~ 0.51)	75.00 (0.22 ~ 0.99)	93.33	14.00	1.33	0.89

### Clinical impact of mNGS on diagnosis and treatment

**Figure 4:**
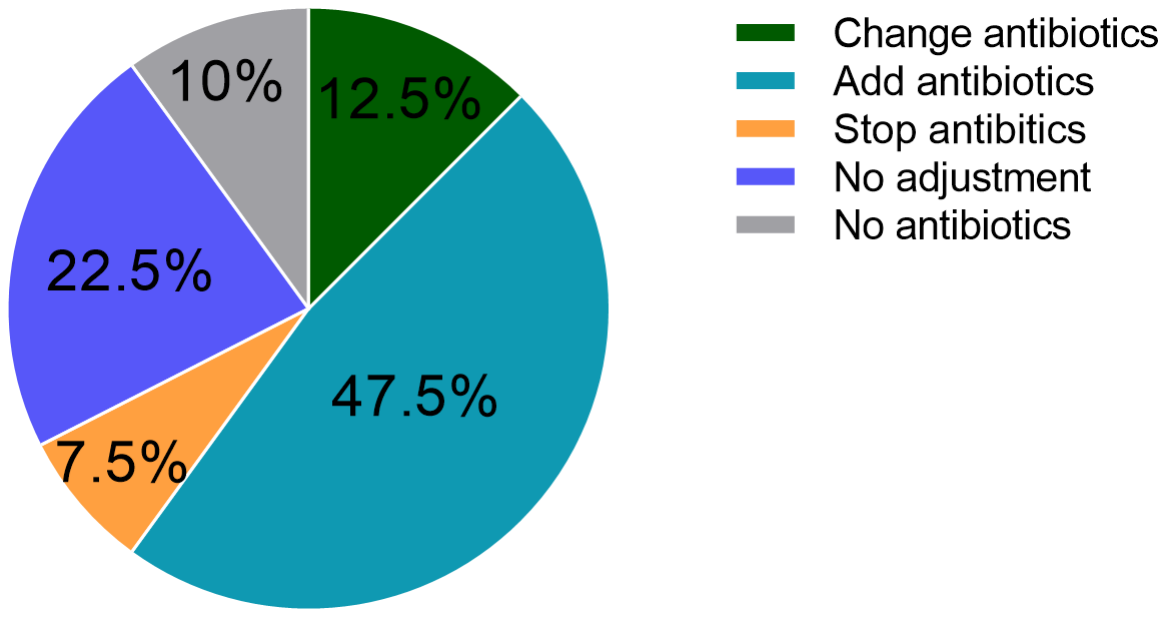
Adjustment of antibiotic drug.

The antibiotic regimen was adjusted based on the mNGS results in 27 (67.5%) of the 40 patients. However, adjustments were abandoned in 9 (22.5%) patients due to compliance with the original antibiotic regimen. Of the 27 cases requiring adjustment of the antibiotic regimen: drugs were added in 19 (70.3%) cases, changes were made in 5 (18.5%) cases, and drugs were discontinued in 3 (11.1%) cases (Figure 4). Sulfamethoxazole was the most commonly adjusted drug before and after the detection of pathogenic bacteria with the mNGS (Table 3). Outcomes were improved in 25 (62.5%) of the 40 patients.

**Table 3:** Comparison of the proportion of antibiotic use before and after adjustment according to mNGS.

Antimicrobials	Pre-mNGS	After-mNGS	*p* value
Voriconazole	5/40 (12.5%)	8/40 (20%)	0.363
Cefoperazone sodium and sulbactam sodium	8/40 (20%)	7/40 (17.5%)	0.775
Piperacillin sodium tazobactam sodium	3/40 (7.5%)	7/40 (17.5%)	0.310
Moxifloxacin	10/40 (25.00%)	10/40 (25%)	1
Cefuroxime	1/40 (2.50%)	0/40 (0.00%)	1
Cefdizine	0/40 (0.00%)	1/40 (2.5%)	1
Fluconazol	2/40 (5.00%)	3/40 (7.5%)	1
Levofloxacin	1/40 (2.5%)	2/40 (5.00%)	1
Sulfamethoxazole	2/40 (5.00%)	12/40 (30.0%)	0.008
Ertapenem	1/40 (2.50%)	1/40 (2.50%)	1
Ceftazidime	0/40 (0.00%)	1/40 (2.50%)	1
Amphotericin	0/40 (0.00%)	1/40 (2.50%)	1
Caspofungin	0/40 (0.00%)	1/40 (2.50%)	1
imipenem and cilastatin sodium	3/40 (7.50%)	2/40 (5.00%)	1
Linezolid	1/40 (2.50%)	2/40 (5.00%)	1
Cefotaxime sodium	0/40 (0.00%)	1/40 (2.50%)	1
Rifaximin	1/40 (2.50%)	1/40 (2.50%)	1
Metronidazole	1/40 (2.50%)	1/40 (2.50%)	1
Meropenem	2/40 (5.00%)	2/40 (5.00%)	1

McNemar test for table.

## Discussion

Infection is the leading cause of death in patients with SARDs, and the lungs are the most common site of infection among those receiving immunosuppressant therapy. However, relatively few studies have investigated the use of mNGS for the analysis of BALF samples from patients with SARDs while receiving corticosteroids or immunosuppressants. The present study aimed to compare mNGS vs. CMTs for the identification of pathogens in BALF samples from patients with SARDs treated with corticosteroids or immunosuppressants. Of the 40 patients, pathogens were detected in the BALF samples of 37(92.5%) by mNGS, 18 (45%) by CMTs, and 16(40%) by both methods, while 1 (2.5%) was negative by both mNGS and CMTs. In addition, co-infections were detected in 31 (77.5%) patients by mNGS as compared to only 4 (10%) by CMTs, which led to adjustments of antibiotic regimens for 27 (67.5%) patients. Notably, symptoms improved in 25 (62.5%) of the 40 patients. By comparing mNGS and CMTs, we found that mNGS had the characteristics of high sensitivity and accuracy in pathogen detection, especially for the diagnosis of fungal, viral, specific pathogenic infections, and co-infection, which helped in clinical decision-making and improved prognosis, and was consistent with the findings of a previous report (19).

Among the 40 patients enrolled in this study, 37 (92.5%) received antibiotic treatment before the collection of the BALF samples. Because the detection rate of CMT pathogens is very low, the test results are uncertain. Because CMT has a low detection rate of pathogens, its detection results are unreliable. The results of CMTs usually require 3–5 days as compared to 1 day for mNGS. Compared to CMTs, the results of mNGS are less affected by antibiotics (20). Meanwhile, a wider spectrum of pathogens can be detected by mNGS, which facilitates diagnosis and treatment timely.

In total, 65 pathogens were identified by mNGS and 19 by CMTs. Most of the identified pathogens were bacteria, and only 3 (4.6%) were fungi. The bacteria identified in BALF samples included Haemophilus influenza, *Streptococcus pneumoniae*, and *Pseudomonas aeruginosa*, in addition to the opportunistic pathogens Trophozoites whipplei and *Prevotella melaninogenica*, which are difficult to detect by CMTs.

The most common pathogens detected by mNGS were CMV, EBV, and P. jirovecii. Of the 15 cases of PJP in this study, 14 (93.3%) were diagnosed by mNGS, and only 1 (6.7%) was confirmed by CMTs. Although PJP is traditionally detected by immunofluorescence staining and PCR, the positivity rate of mNGS for the detection of PJP is superior to that of CMTs (21). Moreover, mNGS was comparatively superior for detection of CMV, EBV, and other opportunistic pathogens. Although less virulent in healthy hosts, opportunistic pathogens can cause severe and frequent infections in patients receiving immunosuppressants (22).

In this study, the detection rate of multiple pathogens by mNGS was superior to that by CMTs (72.5% [29/40] vs. 12.5% [5/40], respectively, *p* < 0.05), demonstrating that mNGS is more suitable for detection of mixed infections (23). Deficiencies of CMTs, such as sensitivity to fungi, viruses, and specific pathogens, in addition to interference by empirical antibiotics can be compensated by mNGS (24, 25).

The treatment regimens of 67.5% (27/40) of patients were adjusted based on the results of mNGS and CMTs, which ultimately resulted in improved outcomes for 62.5% of the cohort. Notably, 10 (25%) patients were diagnosed with PJP based on the mNGS results. Of these cases, the antibiotic regimen was terminated or switched to sulfamethoxazole in 7 (70%). These results suggest that mNGS can more accurately identify pathogens causing pulmonary changes, thereby avoiding overuse of antibiotics.

There were some limitations to this study that should be addressed. First, it’s difficult to obtain BALF from patients with SARDs as a control group when bronchoscopy is not performed in the patient without lesion in the lung. Second, the patients were recruited from a single medical center, which might have introduced bias. Third, it was difficult to determine whether the pathogens detected by mNGS were collateral contaminants, opportunistic colonizers, or causative pathogens because all patients received immunosuppressant therapy for SARDs. Fourth, there may have been some false positives of patients with mixed infections. Therefore, mNGS requires further development to accurately identify multiple types of pathogens. Finally, some patients did not follow up with outpatient clinics or examinations within the prescribed time, resulting in a low follow-up rate. In addition, hospital phone numbers were classified as high-frequency nuisance numbers, making follow-up visits more difficult.

In conclusion, mNGS were significantly better than CMTs for detection of suspected pulmonary infections in patients with SARDs receiving immunosuppressant therapy. Rapid diagnosis by mNGS can ensure timely adjustment of treatment regimens to improve diagnosis and outcomes.

## Data availability statement

The data presented in the study are deposited in the NCBI repository, online at https://www.ncbi.nlm.nih.gov/bioproject/PRJNA979827.

## Ethics statement

The studies involving human participants were reviewed and approved by the Clinical Trials and Biomedical Ethics Committee of Guangdong Provincial People’s Hospital [KY-H-2022-057-03]. The patients/participants provided their written informed consent to participate in this study.

## Author contributions

SW, SLiu, and LX were responsible for the overall design and investigation. SW, SP, and SLiu were responsible for manuscript writing and participated in discussion of the results. XH, NJ, BL, BC, SD, YY, QW, SLi, and YT were responsible for data collection and analysis. JM, SLiu, TL, FW, ZL, RH, and ZF were contributed to the patients follow study. CH, WW, XL, and WS were served as lead illustrator of this manuscript. All authors contributed to the article and approved the submitted version.

## Funding

This study was supported by National Natural Science Foundation of China (Nos. 81870508, 81873616, and 82170730), Science and Technology Planning Project of Guangzhou (Nos. 202102020534 and 20210208040), Guangzhou Municipal Science and Technology Plan Project (Nos. 202102080385 and 201904010026), Natural Science Foundation of Guangdong Province (Nos. 2022A1515012374 and 2023A1515010024), Young Talent Training Program of Guangdong Provincial Association for S&T (No. SKXRC202222), Guangdong Basic and Applied Basic Research Foundation (No. 2021A1515220150), and Guangdong Province High-level Hospital Construction Project (No. DFJH201901).

## Conflict of interest

The authors declare that the research was conducted in the absence of any commercial or financial relationships that could be construed as a potential conflict of interest.

## Publisher’s note

All claims expressed in this article are solely those of the authors and do not necessarily represent those of their affiliated organizations, or those of the publisher, the editors and the reviewers. Any product that may be evaluated in this article, or claim that may be made by its manufacturer, is not guaranteed or endorsed by the publisher.
